# Prevalence of Echocardiographic Evidence of Trace Mitral and Aortic Valve Regurgitation in 50 Clinically Healthy, Young Adult Labrador Retrievers without Heart Murmur

**DOI:** 10.3390/ani12182442

**Published:** 2022-09-16

**Authors:** Maxime V. de Jong, Peter A. J. Leegwater, Hille Fieten, Viktor Szatmári

**Affiliations:** Clinical Sciences, Faculty of Veterinary Medicine, Utrecht University, Yalelaan 108, 3584 CM Utrecht, The Netherlands

**Keywords:** auscultation, congenital, hereditary, physiologic regurgitation, valve dysplasia

## Abstract

**Simple Summary:**

Screening for various disorders lowers the chance for spreading hereditary diseases by selecting individuals for breeding that do not have that specific disorder. Screening examinations, which are typically performed in clinically healthy animals, can pick up diseases in an early stage, when clinical signs are absent. On the other hand, screenings might also reveal anomalies of questionable relevance. In the present study, healthy young Labrador retrievers were screened with cardiac ultrasound for congenital heart defects. In half of the dogs, a small leakage of a cardiac valve was found, which finding is often a sign of a heart disease. However, in this breed and age group, this leaky valve is most likely innocent and represents a variation of normal.

**Abstract:**

**Background—**Though physiologic regurgitation of the right-sided cardiac valves is well recognized in dogs and other mammals, the prevalence of trace insufficiency of the mitral and aortic valves in clinically healthy, young adult dogs is unknown. **Methods—**In this observational cross-sectional study, 50 clinically healthy, young adult Labrador retrievers without an audible heart murmur were enrolled. All dogs were bred and owned by a single organization. Cardiac screening was requested for all dogs that were intended for breeding. These dogs underwent a cardiac auscultation and transthoracic echocardiography by a veterinary cardiology specialist. If mitral or aortic valve regurgitation was noticed, the jet size was subjectively assessed on color Doppler echocardiography. Pedigree analysis was performed to reveal a possible hereditary background of mitral valve regurgitation. **Results—**The prevalence of trivial mitral valve regurgitation was 52% with no significant predisposition to gender (*p* = 0.86) or haircoat color (*p* = 0.68). The prevalence of aortic valve regurgitation was 4%. Pedigree analysis for mitral valve regurgitation showed familial clustering, suggesting a hereditary background of the trait. **Conclusions—**The prevalence of silent trace mitral valve regurgitation in young adult Labrador retrievers was high. Because the regurgitant jet was trivial in all dogs, it is probably physiologic.

## 1. Introduction

Competent cardiac valves play a crucial role in physiological cardiac function. However, color Doppler echocardiography reveals trace to mild tricuspid and pulmonic valve regurgitations so often that these findings are thought to be physiologic in various mammalian species [1,2,3,4,5,6]. As the regurgitant volume of these physiologic regurgitation jets is small, it does not cause an audible murmur, nor right ventricular volume overload. Whether a trace regurgitation of the left-sided cardiac valves, i.e., the mitral and the aortic valves, in the absence of cardiac murmur is also a physiologic finding or represents a mild form of congenital valve dysplasia, in dogs is unknown. This differentiation is especially important if the regurgitation is found at a screening examination in dogs that are intended for breeding purposes. In case of a hereditary congenital heart disease, the offspring could potentially suffer from a more severe valvular regurgitation.

The present study aimed to determine the echocardiographic prevalence of trace mitral and aortic valve regurgitation in young adult, clinically healthy Labrador retrievers without a heart murmur. In addition, a pedigree analysis was performed to reveal a possible hereditary background of these valve regurgitations.

## 2. Materials and Methods

### 2.1. Animals

In this retrospective study, medical records of clinically healthy Labrador retrievers owned by a single dog breeding organization were searched in the electronic database of the authors’ institution between 2015 and 2021. Inclusion criteria were that the dogs had to be between 1.0 and 2.5 years of age, free of clinical signs and free of cardiac murmur at the time of the screening examination. Exclusion criterion was the echocardiographic presence of a concomitant cardiac anomaly.

### 2.2. Examinations

All physical and echocardiographic examinations took place at the request of the dog breeding organization. Therefore, the examinations fell under regular clinical patient care and were not an animal experiment. The aim of the heart screening was to rule out congenital, potentially hereditary, cardiac anomalies as these dogs were selected for breeding purposes.

After a short history taking, cardiac auscultation took place by a veterinary cardiology specialist. Immediately after the auscultation, an echocardiographic examination was carried out by the same specialist. Transthoracic echocardiography was performed on unsedated dogs in right and left lateral recumbency according to reported standards using two-dimensional, M-mode, color Doppler and spectral Doppler modes [5,6]. The mitral valve morphology and the presence of mitral valve regurgitation was assessed with transthoracic two-dimensional and color Doppler mode from the standard right and left parasternal long axis four-chamber views [5,6]. Left atrial to aortic ratio was measured to determine left atrial size, and the normalized left ventricular diastolic internal dimension was measured to determine whether there were echocardiographic signs of volume overload [5,7,8,9]. The presence of mitral valve stenosis was assessed from the standard left parasternal four-chamber view by evaluating the mitral inflow pattern by using pulsed-wave Doppler mode [5,6]. Morphologic evaluation of the aortic valves and the presence of aortic valve regurgitation took place in the standard right parasternal five-chamber long axis and short axis views as well as in the left parasternal views by two-dimensional and color Doppler imaging [5,6]. The presence of aortic stenosis was assessed using the subcostal view by continuous wave spectral Doppler technique [5]. Aortic stenosis was diagnosed when the peak flow velocity exceeded 2.2 m/s [10].

Only the presence or absence of mitral and aortic valve regurgitation was recorded. The jet size was subjectively rated based on the size of color Doppler jet. The jet size of the mitral valve was graded as trivial when it occupied a small area in the left atrium, close behind the closed valve leaflets in systole.

### 2.3. Statistical Analysis

Descriptive statistics were used to describe the characteristics of the study sample. The prevalence of mitral and aortic valve regurgitation was calculated as a proportion of the total number of dogs.

The association of gender and blonde or black coat color to mitral valve regurgitation was analyzed with the Chi-squared test using a commercially available statistical software package (SPSS version 26, IBM©, New York, NY, USA).

## 3. Results

### 3.1. Animals

The electronic search identified 66 pure bred Labrador retriever dogs that were owned by the single dog breeding organization and underwent echocardiography in the study period. From these 66 dogs, a total of 16 dogs were not included: 8 dogs due to the presence of clinical signs, 6 dogs due to the presence of an audible heart murmur and 2 dogs due to clinical indication for echocardiography (i.e., other than screening; 1 dog had intermittent audible third heart sound and the other dog had right bundle branch block on a surface ECG); this resulted in 50 dogs for the study sample. This group of 50 dogs consisted of 14 intact males and 36 intact females with a median age of 14.3 months (range 12.5–27.1 months), of which 16 had black and 34 had blonde haircoat.

### 3.2. Prevalence of Mitral and Aortic Valve Regurgitation

**Figure 1:**
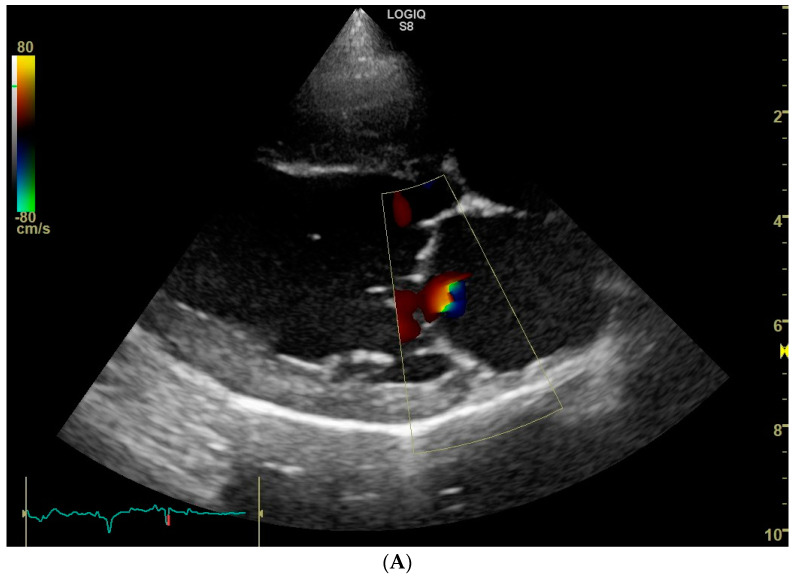

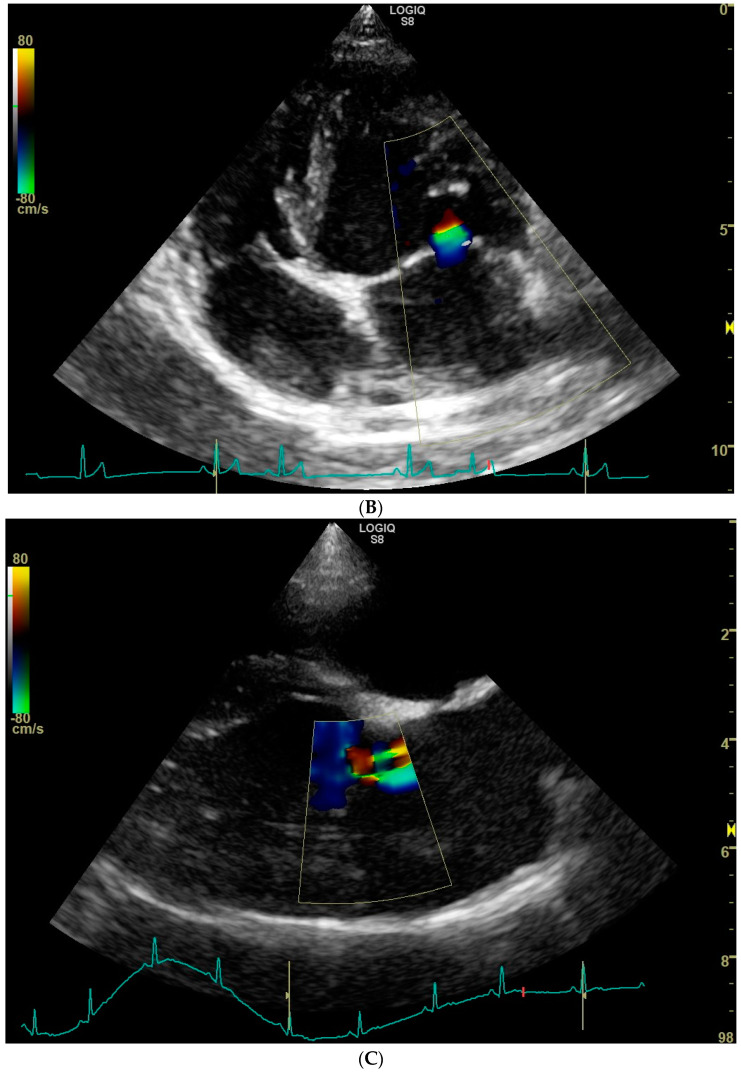
Color Doppler echocardiographic images (systolic frames) of trivial mitral valve regurgitation jets in three clinically healthy Labrador retrievers without heart murmur. (**A**). Standard right parasternal four-chamber view showing a central jet. (**B**). Standard left parasternal four-chamber view showing a central jet. (**C**). Standard right parasternal four-chamber view showing two eccentric jets.

Trivial mitral valve regurgitation was detected in 26 dogs, resulting in a prevalence of 52%. No obvious morphologic changes were noticed on the mitral valve apparatus with 2D echocardiography. Mitral regurgitation appeared central in 24 dogs and eccentric in the remaining 2 dogs, of which in one case two small jets were noticed (Figure 1). Because of the small jet size, no attempts were made to measure the flow velocity of the regurgitant jet with spectral Doppler mode. Mitral valve regurgitation was equally prevalent in female dogs (19/36, 53%) compared to male dogs (7/14, 50%) (*p* = 0.86). Moreover, mitral valve regurgitation was equally prevalent in black and blonde dogs: 9/16 (56%) in the black and 17/34 (50%) in the blonde ones (*p* = 0.68).

Aortic valve regurgitation was detected in two dogs, both without aortic stenosis or ventricular septal defect, and without echocardiographic signs of endocarditis, resulting in a prevalence of 4%. The aortic valves in both dogs were tricuspid and the regurgitant jet was central. No obvious morphologic changes were noticed on the aortic valve with 2D echocardiography. Both dogs with regurgitation of the aortic valve also had mitral valve regurgitation. Aortic valve regurgitation was found in one male and in one female dog, of which one was black and the other one was blonde.

### 3.3. Left Ventricular and Left Atrial Sizes

The diameter of the left atrium and aorta were measured on two-dimensional images according to the “Swedish method” using the standard right parasternal short axis images [5,7,11]. The median left atrial to aortic ratio measured 1.37, ranging from 1.01 to 1.79 (reference < 1.6). Left atrial to aortic ratio of higher than 1.6 was measured in six dogs, all six with mitral valve regurgitation. According to the recently revisited guidelines, a left atrial to aortic ratio of <1.7 can still be considered normal [11]. The left ventricular internal dimension in diastole was measured on M-mode images using the standard right parasternal short axis view and it was corrected to the body weight to calculate the normalized value, resulting in a median of 1.47, ranging from 1.21–1.76 (reference 1.27–1.85) [5,8,9].

### 3.4. Pedigree Analysis

**Figure 2:**
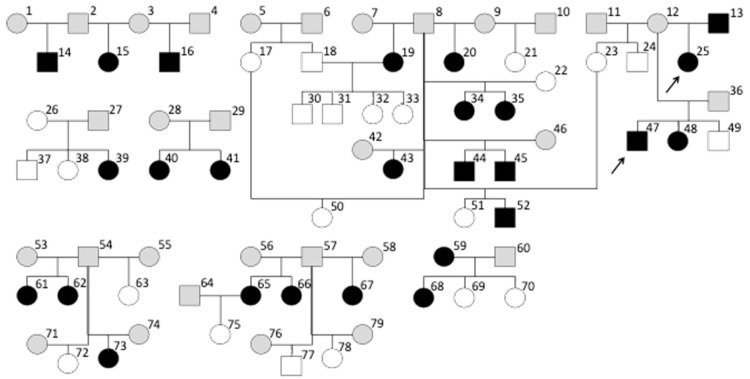
Pedigree lines of 50 Labrador retrievers and their ancestors illustrated by family trees. There is one family (parents #18 and #19 with an offspring #30–33) whose members all underwent an echocardiogram. Dog #18 was a male without mitral regurgitation, while dog #19 was a female with mitral valve regurgitation. None of their puppies had a mitral valve regurgitation. When assuming that a mitral valve regurgitation trait depends on a monogenetic inheritance, the absence of mitral valve regurgitation in the four dogs of this litter raises the suspicion of an autosomal recessive inheritance. Aortic valve regurgitation was documented in dogs #25 and #47 (arrows). They had a common parent (dog #12). Circle = female; square = male; black = mitral valve regurgitation present; white = mitral valve regurgitation absent; gray = presence of mitral valve regurgitation is unknown; arrow = aortic valve regurgitation present.

To investigate the presence of a possible hereditary background of mitral valve regurgitation, the pedigrees of the dogs enrolled in this study were analyzed (Figure 2). Pedigree information was received from the dog breeding organization that owned all the dogs. The enrolled dogs originated from 33 litters. Though not every dog of each litter was screened, mitral regurgitation was found in dogs originating from 20 litters. In 12 litters, more than 1 dog underwent screening. In 9 of these 12 litters, at least one individual had mitral valve regurgitation, and in 6 of these litters multiple affected dogs were identified. Mitral valve regurgitation in these dogs was almost equally distributed in males and females.

Since the parents of the enrolled dogs did not routinely undergo an echocardiogram, the presence of mitral valve regurgitations and heart murmurs in the majority of the parents was unknown. In 7 of the 33 litters, one of the parents underwent echocardiography. There was only one family where both parents and the whole litter underwent an echocardiography. In this family, the farther had no mitral regurgitation and the mother did. The mating resulted in four pups. Echocardiography revealed no mitral valve regurgitation in any of these four dogs.

Due to the low prevalence of aortic valve regurgitation in the study sample, pedigree analysis was not performed for this condition.

## 4. Discussion

The present study found a trace mitral regurgitation in half of the examined clinically healthy, young adult Labrador retriever dogs without heart murmur.

The cause of mitral valve regurgitation could be the result of either an individual variation of normal or a mild mitral valve dysplasia. Mitral valve dysplasia is a relatively uncommon congenital cardiac anomaly in dogs, which represents 2–8% of all congenital cardiac defects in mixed breed dogs and shows a predisposition for males [12,13]. Based on the young adult age of the dogs included in this study, myxomatous mitral valve degeneration can be excluded as a possible cause of mitral valve regurgitations. Infectious endocarditis can also be excluded based on valve morphology, the lack of suggestive medical history and the high prevalence of regurgitation.

There was only one family of which each dog underwent an echocardiography. No sound conclusions about the mode of inheritance, such as the likeliness of monogenetic inheritance or multifactorial inheritance properties, can be drawn based on the available data. The familial clustering of the cases does suggest that the predisposition to the trait is inherited.

There are only few studies on silent (i.e., without audible murmur) mitral valve regurgitation in dogs. One study on 20 clinically healthy, young adult beagles without an audible heart murmur (age range 8–17 months) from 1994 described a prevalence of 15% (3/20) of mitral valve regurgitation and 10% (2/20) of aortic valve regurgitation on color Doppler echocardiography [14]. The true prevalence of these regurgitations might be higher today due to the increased sensitivity of color Doppler echocardiography over the past 25 years. An echocardiographic study on clinically healthy adult English Springer spaniels (with a median age range of 2.5–12.8 years) reported trivial to mild mitral valve regurgitation without specifying the prevalence [15]. In contrast, a more recent echocardiographic study on a large number of clinically healthy Dobermans does not mention mitral valve regurgitations [16]. Another canine study focusing on the relationship between physical activity and echocardiographic changes in English Setters, however, reported mild mitral valve regurgitation in 7 of 100 clinically healthy adult dogs [17]. All these seven dogs were in the “active” group, but only three of them were under five years of age [17]. Even in these older English Setters, where mitral valve degeneration is more likely to be present, the prevalence of mitral valve regurgitation was much lower than in our group of young adult Labrador retrievers. A study on clinically healthy Border Collies is the only report we found that documented a similarly high prevalence (45%) of trace to mild mitral valve regurgitation to our findings [18]. However, the mean age of the enrolled 20 dogs was much higher (6 years, range 2–12 years) than in our study, increasing the likelihood of pathologic valve regurgitation due to acquired valve disease.

Congenital tricuspid valve dysplasia and Ebstein’s anomaly in Labrador retrievers are well-recognized hereditary disorders [19,20,21]. Labrador retrievers are reported to have an increased risk of tricuspid valve dysplasia by 35 times compared to other breeds, and by 7 times compared to mixed breeds [19]. In a kindred of related Labrador retrievers, Ebstein’s anomaly was demonstrated to be a monogenic disease with an autosomal dominant trait with a hereditary factor of 0.71 [21].

Regarding silent mitral valve regurgitation in other species, it has been reported in cats, human beings and horses. A retrospective study describing the prevalence of physiologic mitral valve regurgitation in cats (age range of 6 months to 3.6 years) reported a prevalence of 9% [3]. The same study reported no aortic valve regurgitation.

In a study where human beings without murmur were investigated, echocardiographic evidence of mitral valve regurgitation was present in 45% of 40 children (age range 6–9 years), in 45% of 47 teenagers (age range 10–19 years), in 43% of 41 young adults (age 20–29 years) and in 41% of 39 adults (age range 30–39 years). Aortic valve regurgitation was not detected in any of the subjects [22]. Another study on human beings concluded that exercise could evoke valve regurgitation as it occurred more often in athletes than in similar control subjects [23]. Of 45 athletes and 26 control subjects, mitral valve regurgitation was detected in 69% of the athletes compared to 27% of the control group [23]. Aortic valve regurgitation was detected in 13% of the athletes compared to 8% of the control group [23]. The median age of the athletes was 41 years (range 22–76 years), and that of the control group was 36 years (range 24–64 years) [23]. The dogs examined in our study did not receive any additional physical training. Two studies on human beings suggest that physiological mitral valve regurgitation in young healthy people exists and it could be a result of ‘’flattening of the thorax’’ during growth, which causes malcoaptation of the mitral valve leaflets due to cardiac compression [24,25]. This phenomenon is unlikely to occur in a dog breed as the Labrador retriever, with a relatively deep chest confirmation.

Physiological valve regurgitation is also described in horses. Mitral and aortic valve regurgitation are common findings in horses used for sport. Though the criteria defining physiological valvular regurgitation vary from study to study, authors agree that regurgitant jets that occupy a small area, close behind the valve leaflets and are of brief duration, are clinically irrelevant [4,5,18]. A study investigated valve regurgitations in 526 thoroughbred racehorses [26]. Based on their performance, they were placed into six groups. In these groups mitral valve related murmurs were detected in 7, 10, 13, 18, 19 and 23% per group, whereas echocardiography detected mitral valve regurgitation in 29, 40, 46, 52, 53 and 60% in the same groups [26]. Horses with the highest performance had the highest prevalence of mitral valve regurgitation [26]. In the same groups, murmurs caused by aortic valve regurgitation were found in 0, 0, 1, 3, 4 and 7%, whereas echocardiographic evidence of aortic valve regurgitation was present in 38, 54, 54, 57, 57 and 65% in the groups [26]. This study suggests that valvular regurgitation in most athletes is caused by altered cardiac-loading conditions [26]. The above-described findings suggest that mild mitral and aortic valve regurgitation in mammalian athletes can occur as a consequence of physiologic cardiac adaptations due to training [18,27,28].

Whether the trace mitral valve regurgitation is a physiologic finding, or a manifestation of a mild mitral valve dysplasia in the present study, remains unclear. However, because none of the dogs that underwent an echocardiogram from this dog breeding organization had more than trivial mitral valve regurgitation, mitral valve dysplasia as a possible cause of trace mitral valve regurgitation seems unlikely. As opposed to an assumed physiologic regurgitation, mitral valve dysplasia would likely manifest in various grades of mitral regurgitation. On the other hand, though presumed physiologic mitral valve regurgitations are thought to cause a central jet [5,18], we did identify dogs with an eccentric and more than one jet (Figure 1C).

The limitations of the present study include its retrospective character, the relatively small sample size and the low number of parents (i.e., whole families) that underwent an echocardiographic examination. Future studies to investigate the possible hereditary basis of silent mitral regurgitation in Labrador retrievers could focus on mating dogs where both parents have mitral valve regurgitation and let the whole offspring undergo an echocardiographic examination. Similarly, mating dogs where one or both parents are free from mitral regurgitation and let their offspring undergo an echocardiographic examination could result in valuable data. In case the mitral valve regurgitation in the offspring remains mild, it can still be the result of an individual variation of normal, i.e., this finding would not facilitate the differentiation of mitral valve dysplasia from physiologic regurgitation. The prevalence of trivial mitral valve regurgitation might be much lower in another breed or even in Labrador retrievers with a different origin. Echocardiographic examination of another group of Labrador retrievers with a different genetic background could help to decide whether the findings of the present study are specific for this group of dogs, or whether they could be generalized to this breed. Because trivial to mild regurgitation of the tricuspid and pulmonic valves were considered physiologic, these findings were not documented. Finally, serial follow-up echocardiographic examinations might have revealed the possible long-term cardiac effects of the valve regurgitations.

## 5. Conclusions

Trace mitral valve regurgitation was found with color Doppler echocardiography in 52% of 50 clinically healthy, young adult Labrador retrievers without cardiac murmurs. The familiar clustering suggests a hereditary basis for the trait. No association between the gender or coat color and the valvular regurgitation was found. This coincidental finding might represent a variation of normal.

## Data Availability

The data that support the findings of this study are available from the corresponding author upon reasonable request.
